# Potential Target Receptors for the Pharmacotherapy of Burning Mouth Syndrome

**DOI:** 10.3390/ph18060894

**Published:** 2025-06-14

**Authors:** Takahiko Nagamine

**Affiliations:** 1 Department of Psychiatric Internal Medicine, Sunlight Brain Research Center, Hofu 747-0066, Japan; tnagamine@outlook.com; Tel.: +81-835-25-6610; 2 Graduate School of Medical and Dental Sciences, Institute of Science Tokyo, Bunkyou 113-8510, Japan

**Keywords:** burning mouth syndrome, NMDA receptor, D2 receptor, pharmacotherapy

## Abstract

**Objective:**Burning mouth syndrome (BMS) is a chronic, intractable orofacial pain condition characterized by a burning sensation in the oral mucosa without discernible lesions. The syndrome predominantly affects menopausal and postmenopausal women and is considered a form of nociplastic pain, where the processing of pain stimuli is altered. Given the significant sex disparity, it is crucial to consider underlying neurobiological differences that may inform treatment. This review explores potential pharmacological targets by examining the pathological mechanisms of BMS. **Method of Research**: A narrative review approach was utilized to systematically explore and synthesize literature regarding the pathophysiology of BMS and to identify receptors implicated in the enhancement of sensory transmission and the altered processing of pain stimuli. **Results**: The mechanism of enhanced sensory transmission points to receptors such as TRPV1, P2X3, and CB2 as potential targets. However, considering the nociplastic nature of BMS and its prevalence in women, mechanisms involving altered central pain processing are paramount. Research indicates significant sex differences in glutamate transmission and plasticity within reward-related brain regions. This suggests that the N-methyl-D-aspartate (NMDA) receptor, a cornerstone of glutamate signaling and synaptic plasticity, is a primary therapeutic target. Furthermore, the altered processing of pain and reward, which is a key feature of chronic pain, implicates the brain’s dopaminergic system. A decrease in dopamine D2 receptor function within this system is believed to contribute to the pathology of BMS. Estrogen receptors are also considered relevant due to the menopausal onset. **Conclusions**: Based on the evidence, the most promising targets for pharmacotherapy in BMS are likely the NMDA receptor and the dopamine D2 receptor. The high prevalence of BMS in women, coupled with known sex differences in the glutamate and dopamine pathways of the reward system, provides a strong rationale for this focus. Effective treatment strategies should therefore aim to modulate these specific systems, directly or indirectly controlling NMDE receptor hyperactivity and addressing the decreased D2 receptor function. Further research into therapies that specifically target this sex-linked neurobiology is essential for developing effective pharmacotherapy for BMS.

## 1. Introduction

Burning mouth syndrome (BMS) is a chronic and intractable orofacial pain syndrome characterized by the presence of a burning sensation on the oral mucosa in the absence of any specific oral lesions. Individuals with BMS experience a constant or intermittent burning, scalding, or tingling pain sensation of unknown cause that can last for months or years, severely reducing their quality of life [1]. This sensation most commonly affects the tongue, but can also occur on the lips, palate, or throughout the entire oral cavity. Patients often describe the feeling as similar to having scalded their mouth with hot coffee. Beyond the burning sensation, many individuals also report other sensory disturbances, such as a sensation of a dry mouth (xerostomia), despite normal salivary gland function, and altered taste perception (dysgeusia), often described as a bitter or metallic taste.

A key characteristic of BMS is that the pain often has a distinct daily pattern. It is typically absent or mild upon waking in the morning, gradually intensifies throughout the day, and peaks in the evening, which can interfere with sleep. The pain may be temporarily alleviated by eating or drinking. In accordance with the International Classification of Orofacial Pain (ICOP), it is categorized as idiopathic orofacial pain, exhibiting neither morphological alterations nor evident macroscopic nerve damage [2]. BMS occurs significantly more frequently in women than in men, with ratios reported as high as 7:1 [1,3]. The average age of onset is typically in the perimenopausal or postmenopausal period, between 50 and 70 years old. While the exact cause of BMS remains elusive, it is believed to involve a complex interplay of factors, including neuropathic changes at the peripheral or central level, hormonal fluctuations associated with menopause, nutritional deficiencies (such as low levels of B vitamins, zinc, or iron), and psychological factors like anxiety, depression, and stress [3].

The primary goal of treatment for BMS is to manage symptoms and improve the patient’s quality of life. Treatment approaches may include medications such as low doses of antidepressants, anticonvulsants, topical therapies like capsaicin cream, and cognitive behavioral therapy (CBT) [4]. Experimental treatments include low-level laser therapy, repetitive transcranial magnetic stimulation (rTMS), and neuromodulation techniques such as spinal cord stimulation or deep brain stimulation [5]. Of these treatments, pharmacotherapy is the most frequently used and accessible. However, since the pathology of BMS is not fully understood, it is sometimes not clear which receptors are targeted by pharmacotherapy. In this article, we present the currently considered pathophysiology of BMS and discuss potential target receptors for pharmacotherapy.

## 2. Research Method

A narrative review approach was utilized to systematically explore and synthesize the literature regarding potential target receptors for pharmacological treatment in BMS. Our search, conducted across PubMed, Web of Science, and Scopus for the period 2010–2024, specifically focused on articles containing the keywords ‘burning mouth syndrome’, ‘mechanism’, ‘pharmacotherapeutic agent’, and ‘receptor’ to identify relevant mechanistic and therapeutic insights. To ensure relevance, we only included peer-reviewed articles published in English. We specifically focused on studies that focused on pharmacological treatments for BMS. We excluded articles that focused primarily on herbal medicines, immunotherapy, or psychological therapies. The identified papers were thoroughly read to extract key findings, theoretical frameworks, and practical implications. The extracted information was then synthesized into the pathology of BMS and its associated receptors. This iterative process led to the identification of target receptors for the pharmacotherapy of BMS, which formed the structure of the discussion. We decided to present the pathology of BMS, examine the associated neural circuits, and identify target receptors for pharmacological therapy.

## 3. Possible Biological Basis of Burning Mouth Syndrome

The biological basis of BMS likely involves a complex interplay of peripheral and central nervous system mechanisms, with hormonal and psychological factors potentially contributing to the condition. These pathological conditions are thought to interact in complex ways and alter the operational processes of sensing and signaling pain. BMS is often characterized by central sensitization, a condition where the nervous system becomes hypersensitive to sensory input. In the brain, this can lead to amplified pain signals, even in the absence of a clear peripheral cause [6].

The pathological classification of pain has recently been divided into three distinct categories: nociceptive pain, neuropathic pain, and nociplastic pain [7]. BMS is mainly classified as nociplastic pain due to the absence of morphological changes in the oral cavity, nociceptor stimulation, or evident neuropathies [8]. Nociplastic pain is defined as pain that arises from altered nociception despite the absence of clear evidence of actual or threatened tissue damage, causing the activation of peripheral nociceptors or evidence for disease or lesion of the somatosensory system, causing the pain [9]. The exact biological mechanism of nociplastic pain remains unclear, but it is believed to involve either enhanced transmission of sensory stimuli in the spinal cord or trigeminal ganglion and altered processing of these stimuli in the brain [9].

The enhanced transmission of sensory stimuli in the spinal cord or trigeminal ganglion plays a crucial role in the development and maintenance of various pain conditions, particularly those involving heightened pain sensitivity like hyperalgesia and allodynia [10]. Neurons in the dorsal horn of the spinal cord and the trigeminal ganglion, which receive sensory input, become more easily activated. Their response to suprathreshold stimuli is amplified, meaning the same level of input from the periphery leads to a stronger output signal. Enhanced transmission of sensory stimuli in the spinal cord or trigeminal ganglion, driven by central sensitization, represents a maladaptive plasticity of the nervous system that significantly amplifies pain signals and contributes to the development of heightened pain sensitivity and chronic pain states [10].

While the spinal cord and trigeminal ganglion play a significant role in the initial processing and amplification of pain signals through central sensitization, BMS can also arise and be maintained through altered processing of sensory stimuli at the level of large-scale brain networks [11]. This involves a complex interplay of various brain regions and networks that go beyond simple nociceptive pathways. Brain imaging studies in individuals with BMS have shown altered activity in pain processing areas of the brain, such as the thalamus, medial prefrontal cortex (mPFC), anterior insular cortex (AI), and the anterior cingulate cortex (ACC) [12]. In individuals diagnosed with BMS, activation of the AI and ACC, which constitute the salience network, is increased. Furthermore, their connectivity often extends to the mPFC and other areas, suggesting a heightened emotional response to pain and a greater cognitive focus on the pain experience [13]. This can contribute to the suffering and disability associated with chronic pain. The mPFC is involved in the top-down modulation of pain, emotional regulation, and cognitive control [14]. In individuals with BMS, structural and functional changes in the mPFC can impair these regulatory functions, leading to reduced inhibition of pain signals and increased negative emotional states like anxiety and depression, which can further exacerbate pain [15]. The importance of connectivity between the nucleus accumbens (NAc) and the mPFC in motivation and emotion processing is increasingly recognized. This connectivity also plays a crucial role in pain modulation [16]. The NAc, a key component of the basal ganglia, mediates emotional and cognitive processes related to pain and analgesia. The transition to a chronic pain state, which is one of the etiologies of BMS, is intimately associated with the regulation of the mPFC by the basal ganglia, with the NAc occupying a central position within the circuit [16]. Declines in basal ganglia function, including that of the NAc, may be responsible for altered mPFC connectivity with the ACC and AI.

In the subsequent chapter, we will proceed to a discussion of potential target receptors for the pharmacological treatment of BMS, which will be divided into two categories: (1) receptors that enhance the transmission of sensory stimuli, and (2) receptors that modify the processing of pain stimuli. We will also consider why BMS is more prevalent in women from the perspective of sex differences in receptor function.

## 4. Receptors Involved in Enhancing the Transmission of Sensory Stimuli

The abnormal pain transmission in BMS is thought to involve several receptors, primarily those involved in sensory perception. First, there are several possibilities for an increase in receptors that transmit pain sensation, indicating their potential role.

Transient Receptor Potential Vanilloid 1 (TRPV1) is a non-selective cation channel that is activated by heat, capsaicin (the pungent component of chili peppers), and protons (acidity). It is expressed on sensory nerve fibers involved in pain and temperature sensation. Studies have shown an increased density of TRPV1-positive nerve fibers in the oral mucosa of BMS patients [17]. This upregulation of TRPV1 may lead to an increased sensitivity to stimuli that normally wouldn’t be painful, contributing to the burning sensation. Some researchers believe that a small fiber neuropathy in the trigeminal system, with increased TRPV1 expression, plays a significant role in BMS. Interestingly, topical capsaicin is sometimes used to treat BMS, as repeated activation of TRPV1 can lead to a depletion of substance P, a neuropeptide involved in pain transmission, potentially reducing symptoms over time.

P2X purinoreceptors (particularly P2X3) are ligand-gated ion channels activated by extracellular ATP. P2X3 receptors are primarily found on sensory neurons and are involved in nociception and mechano-sensation. Similar to TRPV1, some studies have reported an increased density of P2X3-positive nerve fibers in the oral mucosa of BMS patients. This suggests that ATP, which can be released under various conditions, including even minor tissue damage or stress, might play a role in activating these receptors and contributing to the pain in BMS [18].

Cannabinoid Receptors (CB1 and CB2) are G protein-coupled receptors that are part of the endocannabinoid system, which is involved in regulating various physiological processes, including pain modulation. CB1 receptors are mainly found in the central nervous system, while CB2 receptors are predominantly expressed on immune cells and peripheral nerve terminals. Studies have shown altered expression of cannabinoid receptors in the oral epithelium of BMS patients, with decreased CB1 and increased CB2 expression. These changes in the endocannabinoid system might affect pain perception and modulation in BMS [19].

Subsequently, an exposition shall be conducted on the mechanisms underlying the wind-up phenomenon, which is a process that has been demonstrated to enhance and transmit pain sensation [20]. The wind-up phenomenon refers to the temporal accumulation of pain in the central nervous system, particularly in the spinal cord or trigeminal nucleus in the case of orofacial pain such as BMS. When nociceptive (pain-transmitting) nerve fibers, particularly C-fibers, are repeatedly stimulated, they release neurotransmitters like glutamate into the synaptic cleft in the dorsal horn of the spinal cord or trigeminal nucleus. Glutamate initially binds to α-amino-3-hydroxy-5-methylisoxazole-4-propionic acid (AMPA) receptors on the postsynaptic neuron, causing a small depolarization. With repeated stimulation and sufficient depolarization from AMPA receptor activation, magnesium ions blocking the N-methyl-D-aspartate (NMDA) receptor are expelled. Once unblocked, the NMDA receptor allows a significant influx of calcium ions into the postsynaptic neuron. This calcium influx triggers intracellular signaling pathways that lead to increased excitability of the postsynaptic neuron. This means the neuron becomes more responsive to subsequent stimuli, even if those stimuli are of the same intensity or even weaker. The main receptor involved in the wind-up phenomenon is the NMDA receptor. The NMDA receptor is a glutamate-gated ion channel that plays a crucial role in synaptic plasticity and the central sensitization of pain. While the initial cause of BMS is often unclear, the wind-up phenomenon, mediated by the NMDA receptor in the trigeminal nucleus, is thought to contribute to the chronic nature of the pain. Even if the initial trigger (e.g., nerve damage, inflammation) resolves, the central sensitization established through wind-up can maintain the persistent burning sensation. This may explain why some BMS patients experience ongoing pain despite a lack of identifiable peripheral pathology [21]. Furthermore, alterations in central pain processing and descending inhibitory pathways, which normally counteract wind-up, might also be involved in BMS. Table 1 lists the receptors involved in promoting the transmission of pain stimuli in BMS and indicates the possibility of pharmacological treatment.

## 5. Receptors Involved in Altered Processing of Pain Stimuli

Brain imaging techniques can provide valuable insights into the neural mechanisms underlying BMS. Functional magnetic resonance imaging (fMRI) measures brain activity by detecting changes in blood flow. It can help identify areas of the brain that are overactive or underactive in patients with BMS, particularly those involved in pain processing. Individuals with BMS exhibit structural abnormalities in the brain, including a reduction in gray matter volume in the mPFC, and demonstrate augmented functional connectivity within the medial pain system, which encompasses the mPFC, ACC, and IC [24]. The mPFC has direct projections to the basal ganglia, with the majority directed towards the striatum, which is a constituent of the basal ganglia [25]. Furthermore, there is a pathway that transmits information from the mPFC to the basal ganglia via the thalamus [26]. The basal ganglia are densely populated by dopamine-secreting neurons, which process information from the mPFC and regulate the activity of extensive brain networks. A reduction in the number of neurons in the mPFC results in a decline in the function of the dopaminergic neurons in the basal ganglia. A positron emission tomography (PET) study assessing dopamine D2 receptors within the striatum of individuals with BMS revealed a reduction in D2 receptor activity and diminished endogenous dopamine levels [27]. Studies have shown that there may be a presynaptic dysfunction of the nigrostriatal dopaminergic pathway in BMS. This has led to the exploration of dopamine agonists like pramipexol, which have shown some success in alleviating BMS symptoms [28]. The development of large-scale brain networks that initiate central sensitization in brains with BMS involves decreased D2 receptor function and increased D1 receptor function in the basal ganglia.

**Figure 1:**
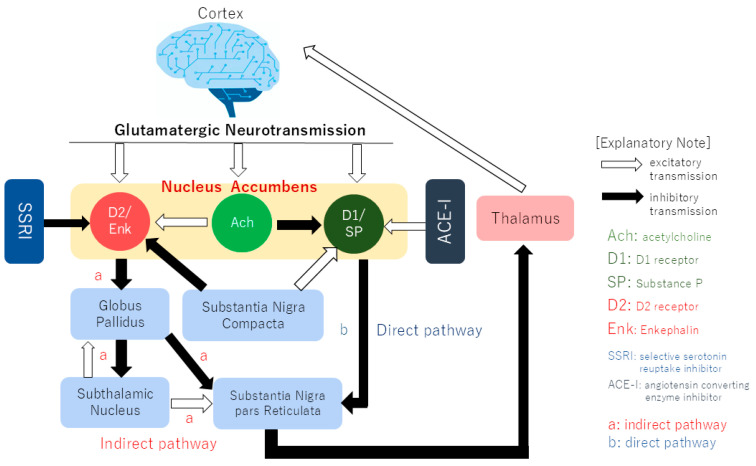
Action points of SSRIs and ACEIs on the dopamine loop. The two drugs, which have different primary pharmacological actions, both demonstrate a mechanism of reducing the output of the dopamine loop in the basal ganglia. Selective serotonin reuptake inhibitors (SSRIs), a class of antidepressants, function by increasing serotonin levels, thereby suppressing dopamine release in the striatum and reducing the output of the dopamine system. This effect is achieved by inhibiting the indirect pathway via D2 receptors. Conversely, angiotensin receptor antagonists, classified as antihypertensive medications, have been observed to activate the D1 receptor coupled with substance P by impeding its breakdown, thereby reducing dopamine output through the stimulation of the direct pathway.

Moreover, clinical observations indicate that dopamine neuron dysfunction in the basal ganglia may contribute to the pathology of secondary BMS. A typical example of secondary BMS is drug-induced. For example, in drug-induced BMS, the causative drug effect is a reduction in basal ganglia dopamine. The most commonly causative medications for drug-induced BMS are selective serotonin reuptake inhibitors (SSRIs) [29] and angiotensin-converting enzyme inhibitors (ACE-Is) [30]. Despite their disparate pharmacological actions, both SSRIs and ACE-Is exert inhibitory effects on the basal ganglia dopamine loop. SSRIs increase serotonin levels and reduce dopamine output by inhibiting D2 receptors via the indirect pathway, whereas ACE-Is stimulate D1 receptors and reduce dopamine output by inhibiting the degradation of substance P via the direct pathway [31]. The administration of both of these drugs has been observed to result in a reduction in dopaminergic neuron output in the basal ganglia dopamine loop (Figure 1). However, in terms of terminology, the term “secondary BMS” is not appropriate. BMS is defined as a chronic pain condition characterized by a burning sensation in the oral mucosa, in the absence of clinically evident mucosal lesions or systemic disease. This definition inherently excludes cases where a specific cause can be identified. If a drug is identified as the cause of burning mouth symptoms, it is no longer a syndrome of unknown origin. Therefore, labeling it “secondary BMS” is contradictory. However, drug-induced burning mouth symptoms (secondary BMS) are crucial for research. They offer a potential window into the underlying mechanisms of BMS. By studying how certain drugs trigger these symptoms, researchers may gain insights into the physiological pathways involved in BMS pathogenesis. The pharmacological action of the drug may represent the pathogenesis of the BMS in these cases. The possibility that these drugs may cause similar pathological changes, such as insular activation in the absence of inhibition from the thalamus to the cerebral cortex, to the morphological and functional changes in the mPFC observed in BMS brains using fMRI is valuable in elucidating the pathology of BMS.

Dopamine neurons in the basal ganglia are connected to two pain-suppressing systems: the reward system and the endogenous opioid system [32]. Consequently, these neurons are involved in the inhibition of pain. In contrast, when dopamine neurons are impaired, the experience of pain is enhanced. The reduction in the mPFC gray matter volume in the brains of individuals with BMS was postulated to induce dopamine dysfunction in the basal ganglia, thereby reducing the analgesic effect of dopamine. In addition to BMS, other well-known chronic pain disorders of unknown etiology, including fibromyalgia, complex regional pain syndrome, chronic fatigue syndrome, and irritable bowel syndrome, have been demonstrated to exhibit diminished mPFC volume [33,34]. A potential commonality among chronic painful diseases is a reduction in neuronal volume within the mPFC, which is accompanied by a decline in dopamine function within the basal ganglia.

## 6. Sex Differences in Receptor Function

### 6.1. Regulation of Receptor Expression by Estrogen

The prevalence of BMS is highest among women aged 65 and over following menopause. Hypotheses suggest that hormonal fluctuations, specifically declines in estrogen, may induce alterations in the nervous system, leading to the manifestation of pain [22]. It is hypothesized that reduced transmission of neuroprotective gonadal hormones via estrogen receptors, which occurs simultaneously with increased stress hormone levels characteristic of the menopausal transition, promotes this process. Moreover, activation and inactivation of estrogen receptors regulate the number of TRPV1 receptors and alter the function of NMDA receptors. While gene expression of the TRPV1 receptor increases during periods of increased estrogen, nerve growth factor (NGF), which incorporates the TRPV1 receptor into cells, also increases during this period. During menopause, a decline in estrogen is accompanied by a concomitant decrease in NGF and an increase in TRPV1 on the cell surface. These findings suggest a dual-stage increase in TRPV1, with estrogen exerting its effect in two phases [35].

Estrogen influences glutamate neurotransmission, and changes in estrogen levels can disrupt the balance of glutamate, the primary neurotransmitter that activates NMDA receptors. A decline in estrogen can lead to increased glutamate release or enhanced NMDA receptor sensitivity. The decline in estrogen removes a regulatory brake on NMDA receptor activity, potentially leading to increased neuronal excitability and contributing to various neurological symptoms [36].

However, despite the correlation between BMS and menopause, hormone replacement therapy (HRT) has not been shown to be effective in treating BMS. A substantial body of research has demonstrated the ineffectiveness of HRT in treating BMS in postmenopausal women [37]. It is believed that years of hormonal fluctuations affect nervous system formation, and estrogen replacement that merely increases estrogen receptor activity does not reduce pain.

### 6.2. Sex-Linked Disparities in Brain’s Reward System

Emerging research reveals that fundamental sex-based differences in the brain’s glutamate system, particularly in regions associated with reward and motivation, may be a key contributor to the higher prevalence of chronic pain conditions in women. These differences appear to be rooted in the function and plasticity of the NMDA receptor, a critical component of glutamate signaling [38]. Glutamate is the primary excitatory neurotransmitter in the brain, essential for a vast array of neural functions, including learning, memory, and synaptic plasticity, the ability of synapses to strengthen or weaken over time. In reward-related brain regions, such as the prefrontal cortex and the nucleus accumbens, glutamate signaling plays a pivotal role in processing pleasure, motivation, and the emotional and affective components of pain. Studies have increasingly pointed to significant sex differences in this system [39]. Preclinical research suggests that females may exhibit higher levels of glutamate transmission and distinct patterns of synaptic plasticity in these reward circuits [40]. This heightened glutamatergic activity can, under certain circumstances, render the system more susceptible to maladaptive changes.

At the heart of these differences lies the NMDA receptor. This receptor is unique in its role as a “coincidence detector”, requiring both glutamate binding and a change in the neuron’s electrical state to become fully active. This property makes it a central player in long-term potentiation (LTP) and long-term depression (LTD), the cellular mechanisms underlying learning and memory, but also chronic pain [41]. Evidence indicates that the function and regulation of NMDA receptors are not uniform between sexes. For instance, the composition of NMDA receptor subunits, such as GluN2B, and their contribution to synaptic plasticity can differ significantly between males and females [23]. Furthermore, female sex hormones, particularly estrogen, have been shown to directly modulate NMDA receptor activity. Estrogen can enhance NMDA receptor function, leading to increased calcium influx into neurons. While this can be beneficial for cognitive processes, it can also lower the threshold for inducing the kind of synaptic strengthening that contributes to the centralization of pain [41].

Chronic pain is increasingly understood not just as a sensory experience but as a condition that profoundly impacts the brain’s reward and emotional circuits. When pain becomes chronic, it can lead to a state of “reward deficiency”, where the brain’s ability to experience pleasure and motivation is diminished. This is thought to be driven by dysregulation of glutamate and dopamine signaling in areas like the prefrontal cortex and nucleus accumbens [40]. The sex-specific characteristics of the NMDA receptor system may make women more vulnerable to this maladaptive plasticity in the face of persistent pain signals [42]. The potentially higher baseline of glutamate transmission and the enhancing effects of estrogen on NMDA receptors could mean that in females, the transition from acute to chronic pain is more readily facilitated at a synaptic level within these crucial reward regions [37]. This could lead to a more pronounced and persistent disruption of reward processing and a greater affective burden of pain. In essence, the very neurobiological factors that may offer advantages in certain cognitive and emotional functions in females could, in the context of injury or inflammation, create a predisposition for the development and maintenance of chronic pain. This line of research underscores the critical need for sex-specific approaches in both the study and treatment of chronic pain, moving beyond a “one-size-fits-all” model to develop more effective and targeted therapies.

## 7. Current Challenges in Receptor-Targeted Pharmacotherapy

Commonly used pharmacological treatments for BMS include antidepressants, clonazepam, and gabapentin. However, the main pharmacological actions of these drugs are different, and the magnitude of their clinical effects is not large.

Antidepressants, particularly amitriptyline, may alleviate BMS by addressing underlying psychological factors and modulating pain pathways through serotonin and norepinephrine. However, the effective doses for BMS are lower than those for depression, and the serotonin paradox highlights the complex role of serotonin, where it can both relieve and exacerbate pain depending on the receptor activated [43]. The efficacy in BMS is likely not solely due to antidepressant effects or descending pain inhibitory pathway activation. Clonazepam, a benzodiazepine, enhances GABA’s inhibitory effects, reducing anxiety and neuronal excitability in both peripheral nerves and the central nervous system. Its effectiveness in BMS seems linked to its impact on pain transmission rather than its anxiolytic or antidepressant properties, potentially acting on peripheral GABA receptors in the oral cavity. Gabapentin, an anticonvulsant, reduces the release of excitatory neurotransmitters like glutamate by binding to calcium channels in the central nervous system. This dampens nerve overactivity contributing to nociplastic pain and modulates pain perception in BMS.

Although these three drugs have different sites of action, they all have mild effects in alleviating the symptoms of BMS, possibly through their modulation of NMDA receptors, one of the receptors underlying BMS pathology. Research suggests that amitriptyline directly antagonizes NMDA receptors [44], while clonazepam indirectly inhibits them by enhancing GABAergic inhibition, and gabapentin reduces their activation by decreasing glutamate release [45]. Amitriptyline functions as an NMDA receptor antagonist through two distinct molecular mechanisms: calcium-dependent desensitization and open-channel block [44]. Amitriptyline has been shown to enhance the calcium-dependent desensitization of NMDA receptors. Consequently, in the presence of amitriptyline, these receptors exhibit diminished responsiveness to glutamate in circumstances where calcium levels are elevated. Additionally, amitriptyline has been demonstrated to function as a trapping open-channel blocker of NMDA receptors. This effect is attributed to the ability of amitriptyline to bind to the receptor’s channel in the open state, thereby effectively impeding the flow of ions through the channel [44]. This common mechanism of reducing NMDA receptor activity may underlie the common therapeutic benefits of these drugs in BMS.

Although control of NMDA receptor function is important for controlling the wind-up phenomenon, it cannot improve the decline in basal ganglia D2 receptor function, which is involved in pain processing. Therefore, it can be pointed out that simply controlling NMDA receptors, which control excitatory transmission, may not be effective enough to treat the pain sensation associated with BMS. This is because the chronification of pain in BMS, like other chronic orofacial pain disorders, is related to a decrease in the function of D2 receptors in the basal ganglia, which are involved in pain processing [46].

## 8. Conclusions

The pathophysiology of BMS was examined to identify target receptors for BMS pharmacotherapy. TRPV1, P2X3, CB2, and NMDA receptors are candidate targets for treating pain transmission in the orofacial region via the trigeminal nerve. D1 and D2 receptors are targets for treating pain formation due to changes in the large-scale brain networks. Decreased estrogen receptor signaling has also been implicated in pain formation. Of these receptors, the NMDA and D2 receptors are thought to be important in the pathogenesis of BMS. Further research is necessary to determine how to effectively control these target receptors for pharmacotherapy.

## Figures and Tables

**Table 1:** Target receptors in the pharmacotherapy of burning mouth syndrome caused by enhancing the transmission of sensory stimuli.

Receptors	Role
TRPV1 receptor [22]	This receptor is upregulated in BMS patients, and its activation leads to pain sensation. Capsaicin, a compound found in chili peppers, is a strong agonist of TRPV1, and its use has shown promise in reducing BMS symptoms.
P2X3 receptor [18]	These receptors are involved in eliciting burning pain sensations, and their activation by adenosine triphosphate (ATP) may play a role in BMS pain.
CB2 receptor [19]	There’s a complex interplay between CB2 receptors and the condition, with studies suggesting an upregulation of CB2 receptors in the tongue of BMS patients. This increase in CB2 expression may be associated with changes in taste perception and pain signaling, potentially contributing to the burning sensation and other symptoms of BMS.
NMDA receptor [23]	Glutamate activates NMDA receptors, triggering a cascade of intracellular events that enhance neuronal excitability and synaptic strength. This phenomenon is sometimes referred to as “wind-up”.

Abbreviations: BMS, burning mouth syndrome; CB2, Cannabinoid Receptor 2; NMDA, N-methyl-D-aspartate; P2X3, P2X Purinoceptor 3; TRPV1, Transient Receptor Potential Vanilloid 1.

## Data Availability

The original contributions presented in the study are included in the article, further inquiries can be directed to the corresponding author.

## Abbreviations

The following abbreviations are used in this manuscript:

AMPA	α-amino-3-hydroxy-5-methylisoxazole-4-propionic acid
ACE-Is	angiotensin-converting enzyme inhibitors
ACC	anterior cingulate cortex
AI	anterior insular cortex
BMS	burning mouth syndrome
CB1	Cannabinoid Receptors 1
CB2	Cannabinoid Receptors 2
CBT	cognitive behavioral therapy
fMRI	functional magnetic resonance imaging
HRT	hormone replacement therapy
ICOP	International Classification of Orofacial Pain
mPFC	medial prefrontal cortex
NGF	nerve growth factor
NMDA	N-methyl-D-aspartate
NAc	nucleus accumbens
PET	positron emission tomography
rTMS	repetitive transcranial magnetic stimulation
SSRIs	selective serotonin reuptake inhibitors
TRPV1	Transient Receptor Potential Vanilloid 1
